# A Case of Sphcelation of the Intestine

**Published:** 1802-05

**Authors:** Robert Jackson

**Affiliations:** Isle of Wight


### To Dr. BRADLEY.

DEAR SIR,

I Enclofe for your Journal, if you judge it of confequence enough to be laid before the public, the cure of Geo. Allifon, which happened under my obfervation while I lived at Stockton upon Tees. I am, &c.

*Isle of Wight, March 29, 1802.* ROBERT JACKSON.

---

### *A Case of Sphacelation of the Intestine, &c.*

GEORGE ALLISON, a failor, (a young man of eighteen of nineteen years of age,) working one day with more than ufual exertion exertion on board of ſhip, was ſeized ſuddenly with a pain in the left ſide, apparently a cramp or ſpaſmodic affection of the muſcles of the abdomen, connected perhaps with ſpaſm of the inteſtine. The ſhip at the time was going out of the harbour, on a voyage to Hamburgh. There was no ſurgeon on board; and conſequently nothing was done for him during the paſſage. He ſuffered great pain; and when the veſſel arrived at her port, his condition was deplorable. A ſwelling was perceivable in the left groin, at the ring of the abdominal muſcles, the uſual place of rupture. It was in fact ſuppoſed to be rupture; and various, and even violent means were employed for the reduction of it; but no object was attained. The lower bowels were emptied frequently by means of glyſters; and ſtrong purgatives were given repeatedly, but without effect. For near ſix weeks, the diſtance of time from the attack, at which he returned to Stockton, and applied for relief at the Diſpenſary, he had no effective evacuation by ſtool. The ſufferings were great, and the caſe appeared ſingular. The belly was not inflated or diſtended generally; but the fleſhy part of the abdomen being waſted, thin, and clinging to the back, permitted the ſmall inteſtines to be traced through their whole extent, knotty and hard, as if crammed with matters which could not find a paſſage onwards. Together with this, there was obſtruction to the act of ſwallowing, connected with a ſenſation at the throat, as if the common motions and progreſs in the alimentary canal met with interruption; the tongue and fauces ſhewed an eryſipelatous redneſs with numerous apthous ſpots; the emaciation of body was extreme; the ſkin was harſh and dry; the pulſe regular, but ſlower than natural, not exceeding 45 in a minute. A ſwelling appeared originally at the ring of the abdominal muſcles on the left ſide. Swelling and inflammation had now extended to the ſcrotum on both ſides, but more particularly on the left, where a fluctuation of matter was evident. The pain of diſtention aggravated the ſufferings; and it was thought adviſable, as a means of affording temporary relief, to give iſſue to this diſtending matter by opening the tumour in the moſt eligible part. This was done accordingly. A large quantity of matter was diſcharged, offenſive to the ſmell, but without appearance of real fæculence. The pain from diſtenſion being thus taken off, a ſhort reſpite and ſome hours of reſt were the conſequence; but there was no change in the ſtate of the original diſeaſe. The difficulty and obſtruction in the act of ſwallowing ſtill remained; and the ſmall inteſtines might ſtill be traced in their courſe by the marks of knotty hardneſs. There was now, at intervals, a growling noiſe in the bowels; and in leſs than thirty hours after the opening opening of the tumour, peafe, bifcuit, and other things, which had been taken in by the mouth for the laft fix weeks, flowed out from the orifice, as from a fluice, with great force, and in quantity fufficient to fill a common chamber-pot. There was alfo about this time a difcharge of urine, which had been fuppreffed for thirty hours or more, and a natural evacuation by ftool, which had not happened for near fix weeks. The diftenfion of the fmall inteftines was now removed; and though the difficulty of fwallowing did not ceafe immediately, nor did the eryfipelatous rednefs and apthous fpots of the fauces immediately difappear, yet there was foon fome defire for liquid food; the pulfe became quicker and more frequent, rifing even as high as 100 in a minute, but without any febrile heat or other fign of fever. The difcharge of fæculent matter continued at intervals, for a few days, through the opening at the groin, accompanied generally with a natural ftool; it diminifhed gradually, and ceafed entirely in lefs than eight days; the natural paffage was reftored, and the evacuations became regular; the eryfipelatous rednefs and apthous fpots of the fauces gradually vanifhed. The fcrotum, which was inflamed through its whole extent, feparated completely on the left fide, from the ring of the mufcle downwards, leaving the tefticle bare, loofe, and dangling, without any fupport but that of its veffels. The general health improved gradually. He recovered his flefh and ordinary appearance; and in the courfe of two months, the tefticle, having been drawn up into the groin, was enveloped in a fpongy covering, which fecured it from injury. At this time there only remained a long puffy rifing, extending upward from the ring of the abdominal mufcle; but there was no complaint of inconvenience, or impediment in the common functions of the parts; this puffy rifing at laft difappeared; and the recovery of health, and of all the functions was fo perfect, that he performed the duties of a common failor; and appears, by late information, to have died in the Weft Indies, in the courfe of the war, on board of a king's fhip.

The cafe of Allifon is perhaps one of the moft fingular upon record. It is now fubmitted to the public; but it is not fubmitted fo directly with the view of conveying practical inftruction, for it contains not much, as of recording the hiftory of an accident, peculiar in its circumftances, and, as far as my knowledge extends, of an event without parallel. The detail is correct. Medical men may be able to find an explanation of confequences fo unufual. I fhall only take the liberty of obferving, that there was probably fome complication of accidents in the cafe, fome preffure, or intus-fufceptio of the gut, near the ring, as well as actual protrufion, the extent of which could could not be afcertained, at the time he applied for relief at the Difpenfary. As a caufe to which the complaint was attributed, he mentions a draught of cold water, which he fwallowed greedily while heated at work; the pain refembling cramp or fpafm in the abdominal mufcles of the left fide, and perhaps in the coats of the inteftine, was the confequence. The violent and harfh means employed for the reduction of a fuppofed rupture, brought inflammation upon the integuments at the ring of the mufcle, and on the fcrotum of the left fide; this inflammation brought in its train adhefion and fuppuration. An abfcefs was formed. It was opened, without any view to ultimate effect upon the original difeafe; but it is probable, that the preffure being thus removed from the protruded inteftine, the rupture of its coats and confequent difcharge of its contents was thereby accelerated: An evacuation by ftool foon followed the difcharge of fæces by the wound in the groin, giving reafon to believe that an intus fufceptio, or fome obftructing preffure upon the gut, muft have alfo taken place; which being now removed by the retrograde motion and difcharge of fæces through the wound, the natural channel was again reftored. The puffy rifing, which remained fome time at the ring of the mufcle on the left fide, after all fymptoms had ceafed, appeared to be owing to dilatation of the inteftine by diftenfion from accumulation of contents, during the obftruction of the paffage. The tefticle was loofe and dangling for fome time, without covering and without fupport. It was doubted, whether or not it would be advifeable to remove it, as it threatened to be an inconvenience; but in a fhort time it was drawn up into the groin, and covered with a fpongy coat, an event which I did not forefee.

